# Cardiopulmonary exercise testing (CPET) in the United Kingdom—a national survey of the structure, conduct, interpretation and funding

**DOI:** 10.1186/s13741-017-0082-3

**Published:** 2018-01-26

**Authors:** T. Reeves, S. Bates, T. Sharp, K. Richardson, S. Bali, J. Plumb, H. Anderson, J. Prentis, M. Swart, D. Z. H. Levett, on behalf of Perioperative Exercise Testing and Training Society (POETTS)

**Affiliations:** 1 Anaesthesia and Critical Care Research Unit, University Hospital Southampton NHS Foundation Trust, Southampton, UK; 2 Critical Care Research Area, National Institute for Health Research Respiratory Biomedical Research Unit, University Hospital Southampton NHS Foundation Trust, Southampton, UK; 3 Integrative Physiology and Critical Illness Group, Clinical and Experimental Sciences, Faculty of Medicine, University of Southampton, Southampton, UK; 4 Department of Anaesthesia and Critical Care Medicine, Plymouth Hospitals NHS trust Hospital, Plymouth, UK; 5 Institute of Cellular Medicine, Newcastle University, Newcastle upon Tyne, UK; 6 Departments of Perioperative and Critical Care Medicine, Freeman Hospital, Newcastle upon Tyne, UK; 7 Department of Anaesthesia and Critical Care Medicine, Torbay Hospital, Torquay, UK

**Keywords:** Cardiopulmonary exercise test, CPET, CPX, Exercise test, Preoperative assessment, Perioperative care, Perioperative medicine, Risk assessment, National survey

## Abstract

**Background:**

Cardiopulmonary exercise testing (CPET) is an exercise stress test with concomitant expired gas analysis that provides an objective, non-invasive measure of functional capacity under stress. CPET-derived variables predict postoperative morbidity and mortality after major abdominal and thoracic surgery. Two previous surveys have reported increasing utilisation of CPET preoperatively in England. We aimed to evaluate current CPET practice in the UK, to identify who performs CPET, how it is performed, how the data generated are used and the funding models.

**Methods:**

All anaesthetic departments in trusts with adult elective surgery in the UK were contacted by telephone to obtain contacts for their pre-assessment and CPET service leads. An online survey was sent to all leads between November 2016 and March 2017.

**Results:**

The response rate to the online survey was 73.1% (144/197) with 68.1% (98/144) reporting an established clinical service and 3.5% (5/144) setting up a service. Approximately 30,000 tests are performed a year with 93.0% (80/86) using cycle ergometry. Colorectal surgical patients are the most frequently tested (89.5%, 77/86). The majority of tests are performed and interpreted by anaesthetists. There is variability in the methods of interpretation and reporting of CPET and limited external validation of results.

**Conclusions:**

This survey has identified the continued expansion of perioperative CPET services in the UK which have doubled since 2011. The vast majority of CPET tests are performed and reported by anaesthetists. It has highlighted variation in practice and a lack of standardised reporting implying a need for practice guidelines and standardised training to ensure high-quality data to inform perioperative decision making.

## Background

Cardiopulmonary exercise testing (CPET) provides an objective, non-invasive measure of functional capacity (ATS/ARCP, 2003). CPET is an exercise stress test with concomitant expired gas analysis. Expired tidal volumes, oxygen and carbon dioxide concentrations, heart rate and respiratory rate are measured and a number of metabolic, ventilatory, gas exchange and cardiovascular variables are derived (ATS/ARCP, 2003). Since Older et al.(1999) first demonstrated that a lower anaerobic threshold was associated with increased mortality in elderly patients undergoing intra-abdominal surgery, more than 30 published case cohort studies have reported that CPET predicts postoperative morbidity and mortality (Wilson et al. 2010; Snowden et al. 2010; Carlisle and Swart 2007; Moran et al. 2016). Consequently, CPET is increasingly forming part of the preoperative assessment. It provides an individualised risk assessment that is used to apprise the decision to proceed to surgery, to inform collaborative decision-making and patient consent, to triage patients to the appropriate level of care perioperatively (e.g. critical care vs surgical ward care), to guide intraoperative anaesthetic techniques, to optimise medical comorbidities preoperatively, to diagnose unexpected comorbidity and increasingly to direct individualised preoperative exercise programmes (prehabilitation) (Older and Levett 2017; Levett and Grocott 2015).

Two previous surveys in England have reported that the use of CPET preoperatively is increasing with the number of trusts offering a service rising from 30 in 2009 to 53 in 2011 (Huddart et al. 2013; Simpson and Grocott 2009). With the implementation of a new diagnostic or prognostic test, it is important to establish whether consistent standards of practice are employed. Valid and reproducible results are vital if the test is used to inform the decision to proceed to surgery, the consent process, preoperative optimisation and the location of perioperative care (e.g. critical care vs ward care). We aimed to evaluate how CPET services have evolved across the UK, to identify who is performing the tests, which patients are being tested, how the tests are performed and interpreted and the funding of CPET services within the NHS.

## Methods

Contact details for all NHS trusts in the UK were obtained from the NHS website (NHS Authorities and Trusts, n.d.). We contacted trusts to establish whether they performed adult elective surgery and identified 197 such trusts. The anaesthetic department was contacted by telephone in each trust and asked if they had a CPET service and to provide details of their CPET and pre-assessment leads. Trusts were telephoned repeatedly until a full list of contact details was obtained.

A structured questionnaire was subsequently sent to the identified service leads. This structured questionnaire was designed using an online survey tool, comprising 211 questions with 4 response arms: trusts with CPET, trusts without CPET, trusts setting up CPET and trusts who had tried but failed to set up CPET (Survey Monkey, n.d.). It contained primarily multiple-choice questions with free text where appropriate. It was not compulsory to answer all question stems of the survey, and some questions permitted respondents to select more than one response.

Questions were written to establish the following:

1. How many centres are performing CPET
2. The types of patients being tested
3. The protocols and equipment being used
4. The methods used for physiological interpretation of the anaerobic threshold
5. Who is performing and reporting the tests
6. The information given in the CPET report
7. Obstacles to CPET
8. The funding model

The online survey was sent to each contact email address in November 2016 with reminders sent to non-responders until March 2017. Data was collected by the online tool and extracted directly for analysis using Microsoft Excel 2011 Version 14.7.0 (Microsoft Corporation, Redmond, WA USA).

## Results

### Response rates to telephone and online survey

We telephoned all 197 anaesthetic departments to obtain an email address for the pre-assessment service leads and a response to the question ‘do you have a CPET service?’ Subsequently, 73.1% (144/197) of these service leads responded to the online survey. It was not compulsory to provide a response to all the questions in the survey, and consequently, the response rates to individual questions varied. Additionally, where appropriate, more than one response could be selected, e.g. referral sources for CPET tests. In such cases, results are reported as the absolute number and the percentage of hospitals that selected this response.

### UK CPET availability

**Fig. 1:**
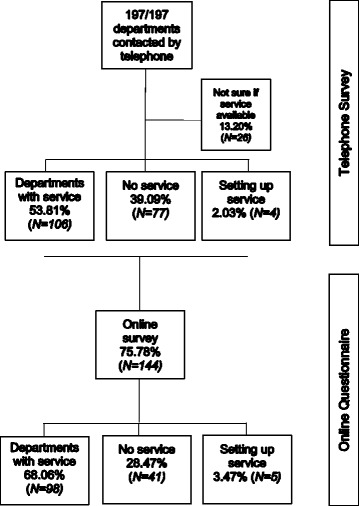
Response rates to survey

Of the 197 trusts performing elective adult surgery contacted by telephone, 53.8% (106/197) have a CPET service, 2.0% (4/197) are in the process of setting up a service, 39.1% (77/197) had no service and 13.2% (26/197) departments contacted were unable to provide an answer (Fig. 1).

### Online survey results

#### Departments with a CPET service from online survey (*N* = 98)

Of the trusts who responded to the survey, 68.1% (98/144) reported that they had a CPET service. Not all respondents responded to every question; consequently, the denominator below reflects the number of responses to the individual question reported.

#### Which patients are tested and how are they selected?

**Fig. 2:**
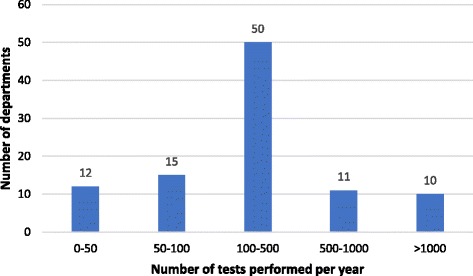
How many tests are performed annually?

**Fig. 3:**
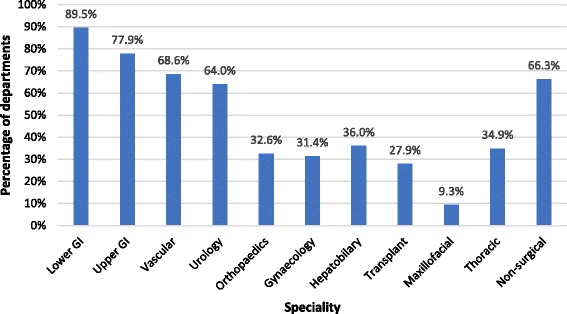
What specialities are tested?

The majority of centres, 50 out of 98 (58.1%), are performing between 100 and 500 tests per year (Fig. 2). Although a wide range of specialities are utilising CPET testing, the most commonly tested group are lower GI with the majority of centres surveyed testing colorectal patients (89%) (Fig. 3). Of note, non-surgical patients are tested in 66.3% of centres. The majority of referrals are made by anaesthetists 84.7% (83/98) and surgeons 83.7% (82/98) with additional referrals from physicians 58.1% (57/98), MDTs 40.1% (40/86), nurse specialists 34.7% (34/98), private hospitals 8.2% (8/98) and other trusts 31.6% (31/98). Patients are primarily selected based on surgery type 75.6% (65/98). Additional factors used to determine who to test include clinical concern 83.7% (72/86), age 16.3% (14/86), screening questionnaire 5.8% (5/86), risk score 7.0% (6/86) and/or other 18.6% (16/86).

#### Where are CPET clinics located?

CPET is performed within the trust in 96.5% (83/86) of centres with the remaining 3.5% (3/86) performed at a neighbouring trust. Clinics are located in pre-assessment 39.5% (34/86), respiratory clinic 24.4% (21/86), cardiology clinic 16.3% (14/86), anaesthetic department 5.8% (5/86) or elsewhere 14.0% (12/86).

#### CPET test conduct: consent and exercise protocol

The majority of centres provide written information about the CPET before the day of the test (61.6% (53/86)). Consent is gained on the day of testing in 90.7% (78/86) of centres. This was verbal in 69.8% (60/86) centres and written in 20.1% (18/86) centres.

**Fig. 4:**
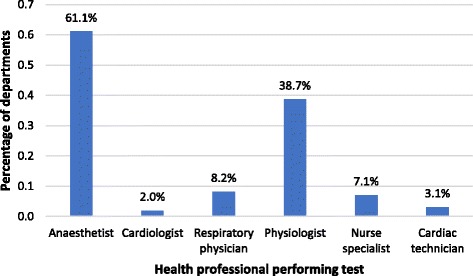
Who is performing CPET tests?

The majority of CPET tests are performed by anaesthetists 69.0% (60/87) and physiologists 43.7% (38/87), but a variety of other clinicians are involved including cardiologists 2.3% (2/87), respiratory physicians 9.2% (8/87), nurses 8.0% (7/87) and cardiac technicians 3.4% (3/87) (Fig. 4). A physician was present for the conduct of all tests in 65.5% (57/87) of centres, for high-risk tests only in 28.7% (25/87) and never in 4.6% of centres (4/87). The additional staff present during the CPET test varied widely and included staff nurses 11.5% (10/87)), health care assistants 19.5% (17/87), cardiac technicians 31.0% (27/87) or other 29.9% (26/87).

Bicycle ergometry is the primary exercise modality used in 93.0% (80/86) of centres, with a treadmill being used in one centre 1.2% (1/86). A hand crank is used when the patient is unable to cycle in 4.7% (4/86) centres. The shuttle walk test is also used in 1 centre (1.2%).

#### CPET test reporting: physiological interpretation and risk reporting

Tests are primarily reported by anaesthetists 73.3% (63/86) although other clinicians also report tests including physiologists 10.5% (9/86), cardiologists 4.7% (4/86), respiratory physicians 4.7% (4/86) and other 7.0% (6/86). In the majority of services, anaerobic threshold is determined by three-point confirmation (V-slope, ventilatory equivalents for VO_2_ and end-tidal O_2_) 86% (74/86). The modified V-slope method is used alone in 6.9% (6/86), the V-slope alone used in 2.3% (2/86), the automated AT generated by the software is used by 8.1% (7/86) and ‘other’ in 11.6% (10/86).

The majority of reports make recommendations about the perioperative care of the patient. These recommendations include the suitability for the proposed operation 77.9% (67/86) (i.e. whether appropriate or alternative procedures or treatment options should be considered), the type of postoperative care 70.9% (61/86) (i.e. elective critical care vs ward), the risk of the procedure 40.7% (35/86), suggestions for preoperative exercise training 33.7% (29/86) and suggested referrals for optimisation 66.3% (57/86) of identified pathology. In 10.5% (9/86), the report only consists of exercise variables. The recommendations are based upon AT 93% (80/86), Peak VO_2_ 86.0% (74/86), VE/VCO_2_ 84.9% (73/86), VE/VO_2_ 19.8% (17/86) and other 44.2% (38/86). Other risk information provided include life expectancy 15.1% (13/86), POSSUM (physiological and operative score for enumeration of mortality and morbidity) mortality 10.5% (9/86), POSSUM morbidity 12.8% (11/86), revised cardiac risk index 16.3% (14/86), National Surgical Quality Improvement Program (NSQIP) 17.4% (15/86), and Surgical Outcome Risk Tool (SORT)15.1% (13/86), and 43.0% (37/86) provide no other risk information.

#### Data quality, training, validation of test results

To ensure data quality, routine validation of test reporting is performed internally in 49.4% (43/87) of centres and internally and externally in 3.4% (3/87) and is not routinely performed in 35.6% (31/87). The majority of departments 96.5% (83/86) agreed that training should be standardised for clinicians reporting CPET. 87.2% (7/86) stated they would consider pursuing standardised accreditation via the Perioperative Exercise Testing and Training Society (POETTS) http://www.poetts.co.uk. 94.2% (82/87) stated they would consider contributing to a national database of CPET data with outcomes held at the Royal College of Anaesthetists Health Services Research Centre to inform risk thresholds.

#### Funding

**Fig. 5:**
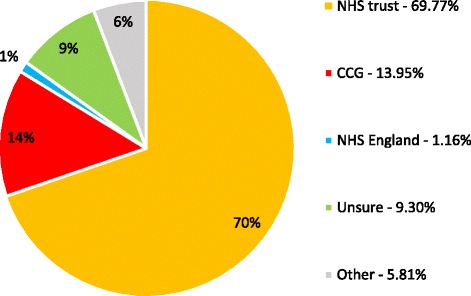
Funding source for CPET services in the UK

The service is funded by a per test fee from clinical commissioning groups (CCG) in 36/86 (41.9%) of services and NHS England in 1/86 (1.2%) of services. The NHS trust funded the service in 41.9% (36/86) trusts. Funding sources were unknown in 8/86 (9.3%) or other in 5/86 (5.8%) (Fig. 5). Where consultant staff are performing the CPET tests, this is considered as programmed activity time in 65.5% (57/87), supporting professional activities (SPA) in 1.1% (1/87) and is unpaid in 5.7% (5/87). Where consultant staff report tests, this is considered as programmed activity time in 64.3% (56/87) and SPA in 8.0% (7/87).

#### Departments without a service (*N* = 41)

58.5% (24/41) of departments without a CPET service cited a lack of funding as the reason; 43.9% (18/41) cited a lack of clinical need, 31.7% (13/41) a lack of staff and 25.0% (10/40) insufficient evidence of benefit. 29% (12/41) of hospitals without a CPET service had previously attempted to set one up. The reasons cited for a previous failed attempt to set up service were an inability to gain finance 91.7% (11/12), lack of clinical justification 58.3% (7/12), administrative problems 16.7% (2/12) and concerns over safety 8.3% (1/12). 83.3% (10/12) reported they would try and establish a service in the future.

## Discussion

We have found that in excess of 30, 000 preoperative CPET tests are being performed annually in the UK and that the number of preoperative CPET services has doubled since 2011 (from 53 to 106). The scope of CPET practice has evolved and expanded since 2011 with more testing in patients undergoing thoracic, urology, hepatobiliary and gynaecology surgery. Furthermore, CPET results are increasingly used to direct preoperative exercise programmes. This may reflect the evolving evidence base supporting its predictive role and the role of prehabilitation (Older and Levett 2017). We have also established that preoperative CPET services are increasingly involved in diagnostic CPET for other specialities.

Hospitals without a CPET service and hospitals who had tried to set up service but failed cited lack of funding as the most common reason. This was also the most frequent response in *Huddart’s* 2011 survey, reflecting continued financial constraints in the NHS (Huddart et al. 2013). There has however been progress in the funding since 2011 with more than 40% now receiving fees from clinical commissioning groups. Furthermore, the majority of clinical sessions are now funded as clinical activity in clinicians’ job plans.

The vast majority of preoperative CPET is performed and interpreted by anaesthetists, with support from a variety of allied health professionals and other physicians. Patients are primarily being selected for testing on the basis of the proposed surgical procedure, but perceived risk and comorbidities are also taken into account. Reporting in the majority of cases involves both physiological variables and advice about the perioperative management and risk stratification of patients. There is increasing focus on preoptimisation by medical referral or exercise interventions compared with previous surveys (Huddart et al. 2013; Simpson and Grocott 2009). This may reflect the growing evidence base supporting prehabilitation prior to surgery (West et al. 2015; Barakat et al. 2016; Barberan-Garcia et al. 2017).

We have identified considerable variability in the conduct of CPET with regards to consent and medical supervision. Given the rare but potentially significant adverse events associated with CPET (e.g. arrhythmias or exercise-induced ischaemia), it would seem appropriate that a formal consent process is followed and medical supervision standardised when the test is performed by a non-medical personnel. This is the case in other areas of clinical CPET practice (Myers et al. 2014).

The anaerobic threshold (AT) is cited as one of the most important variables for advising on risk given its predictive utility in the CPET literature (Moran et al. 2016). The AT can be used to identify a patient population at high risk of postoperative morbidity and mortality. The threshold value used to delineate this high risk patient population varies with the surgical procedure and has been summarised in recent reviews (Moran et al. 2016; Older and Levett 2017). There is known inter-observer variability in determination of the anaerobic threshold (Sinclair et al. 2009; Myers et al. 2010). Three-point determination of the AT, using the V-slope or modified V-slope method with confirmation from the ventilatory response to exercise, is the most reliable and valid approach for AT determination (Ward 2007). Although the majority of CPET services used this approach, this was not consistent. Of concern, in 8% of services, the automated AT generated by the software was used to determine the AT. The exact methods used for automated AT detection in commercial CPET systems vary, but are based on linear regression of the VO_2_-VCO_2_ relationship—the V-slope method. Automated ATs should be interpreted with caution. The manufacturers recommend that they are used to support clinician identification of the AT and that they should not be used in isolation. The kinetic phase at the start of the ramp, and data above the respiratory compensation point must be excluded from the regression analysis, which requires manual interrogation of the data. Furthermore, in the presence of a curvilinear $\dot{V}{CO}_2$-$\dot{V}{O}_2$ relationship or very noisy respiratory data (for example in the presence of significant lung disease), linear regression may not accurately identify the AT. If data is to be compared between centres, standardised interpretation is important. There is an appreciation of the importance of standardisation in the preoperative community as 96.5% of those surveyed supported the introduction of standardised training for CPET practitioners.

A variety of other variables is also being used to evaluate perioperative risk and to contribute to informed consent and shared decision-making. These include the peak VO_2_ and VE/VCO_2_ reflecting the CPET evidence base (Older and Levett 2017). In some services, physiological variables are reported alone; in others, the CPET data is used in combination with life expectancy data and other perioperative scores (POSSUM (Copeland et al. 1991), Lee Revised Cardiac Risk Index (RCRI) (Lee et al. 1999), National Surgical Quality Improvement Programme (NSQIP) (Khuri et al. 1998), Surgical Outcome Risk Tool (SORT) (Protopapa et al. 2014; Wong et al. 2017)), to provide a more comprehensive risk evaluation and inform perioperative care. The approach used is not consistent across centres. This may reflect the different organisational structure of CPET services within the perioperative period. In some centres, CPET forms part of a high-risk pre-assessment or shared decision-making clinic, run by anaesthetists, and the report incorporates a comprehensive risk analysis and plan for the perioperative period reflecting an integrated approach to comprehensive perioperative care. In other centres, CPET functions more like an external referral to a separate team with a report of the physiological variables produced by the CPET team which is subsequently used by the perioperative team at pre-assessment to contribute to the comprehensive risk assessment. If the latter approach is taken, it is important that the perioperative team are expert in the risk implications of the physiological data in the CPET report and the relevant risk thresholds. Equally in order to contribute to preoperative decision-making, it is important that the test is performed sufficiently early in the patient pathway to permit preoperative optimisation (Grocott et al. 2017).

In order to ensure consistent data quality, it is essential that CPET equipment is regularly maintained and serviced. In addition, validation of reporting by internal and external review is important to ensure reporting quality. Only half of the centres surveyed performed internal validation with other clinicians within the service reviewing results, and 3% performed external validation. The low rate of external validation is probably affected by the absence of a national network of perioperative CPET centres to support the exchange of data between groups and external review. Of note, 87.2% of centres suggested they would support establishing a national network of perioperative CPET centres on the national society for perioperative exercise testing and training website (POETTS, http://www.poetts.co.uk) to facilitate peer support and mentoring. As a consequence, a peer network has been established by POETTS and will imminently be available on the website. Recommendations for internal and external validation have been made in recently published perioperative CPET clinical guidelines (Levett et al. 2018).

The evidence base supporting perioperative CPET is currently largely retrospective and single centre (Older and Levett 2017) although multicentre analyses have recently been published (Carlisle et al. 2015; West et al. 2016). Recent systematic reviews have made recommendations about risk thresholds for major surgical patients that are based on results from surgical cohorts from the 1980s to the present day and may not reflect current practice (e.g. data predates the advent of laparoscopic surgery) (Moran et al. 2016). The validity of these historical thresholds for current practice is questionable. There is an urgent requirement to identify contemporaneous risk thresholds. Our survey has revealed the very high volume of tests being performed annually in the UK. There is consequently the opportunity to collect CPET and outcome data nationally to inform risk thresholds based on recent, local data. The aim of any such initiative should be to provide procedure-specific, contemporaneous risk thresholds and information to inform perioperative practice. The database should contain CPET variables, procedure details, complications, length of hospital and critical care stay and mortality. It should link with other perioperative quality improvement datasets that are being collected such as PQIP (perioperative quality improvement programme at the Health Services Research Centre, Royal College of Anaesthetists) (Perioperative Quality Improvement Programme at the Health Services Research Centre, Royal College of Anaesthetists, n.d.). 95.3% of those who responded to the survey stated they would consider contributing to a national CPET database suggesting there is a desire for such a project within the CPET community.

The survey has some weaknesses. Responses to the online survey were incomplete in some cases as we did not make the answer to all questions mandatory. Its strengths are that it is the first survey to comprehensively cover all acute trusts in the United Kingdom. Furthermore, the scope of the survey was broadly covering the structure, conduct, reporting and funding of CPET nationally. We had a complete response to our telephone survey, and our response rate to the subsequent online survey was higher than the previous two surveys. This may reflect persistence with email and telephone reminders to the participants.

## Conclusion

This survey has identified the continued expansion of perioperative CPET services in the UK which have doubled since 2011, testing more than 30,000 patients annually. The vast majority of CPET tests are performed and reported by anaesthetists. It has highlighted variation in practice and a lack of standardised reporting. This suggests that there is a need for practice guidelines and standardised training to ensure high-quality data to inform perioperative decision-making. The recently published perioperative cardiopulmonary exercise testing practice guidelines should support standardising practice (Levett et al. 2018). A national CPET database would provide a means of generating contemporaneous risk data and support peer networking for external CPET report validation and there is support for this from the preoperative CPET community.

## Abbreviations

AT: Anaerobic threshold
CPET: Cardiopulmonary exercise testing
NHS: National Health Service
Peak VO_2_: Peak volume of oxygen consumed
POETTS: Perioperative Exercise Testing and Training Society; CCG: clinical commissioning groups
SPA: Supporting professional activities
UK: United Kingdom
VE/VCO_2_: Minute ventilation/carbon dioxide production
