# Correction to: Cardiopulmonary exercise testing (CPET) in the United Kingdom—a national survey of the structure, conduct, interpretation and funding

**DOI:** 10.1186/s13741-018-0087-6

**Published:** 2018-05-04

**Authors:** T. Reeves, S. Bates, T. Sharp, K. Richardson, S. Bali, J. Plumb, H. Anderson, J. Prentis, M. Swart, D. Z. H. Levett, on behalf of Perioperative Exercise Testing and Training Society (POETTS)

**Affiliations:** 1 Anaesthesia and Critical Care Research Unit, University Hospital Southampton NHS Foundation Trust, Southampton, UK; 2 Critical Care Research Area, National Institute for Health Research Respiratory Biomedical Research Unit, University Hospital Southampton NHS Foundation Trust, Southampton, UK; 3 Integrative Physiology and Critical Illness Group, Clinical and Experimental Sciences, Faculty of Medicine, University of Southampton, Southampton, UK; 4 Department of Anaesthesia and Critical Care Medicine, Plymouth Hospitals NHS trust Hospital, Plymouth, UK; 5 Institute of Cellular Medicine, Newcastle University, Newcastle upon Tyne, UK; 6 Departments of Perioperative and Critical Care Medicine, Freeman Hospital, Newcastle upon Tyne, UK; 7 Department of Anaesthesia and Critical Care Medicine, Torbay Hospital, Torquay, UK

## Correction

After publication of this article (Reeves et al. 2018), it was noticed that two references to the data contained within Fig. 5 were incorrect. In the ‘Funding’ section of the ‘Results’, the line that reads, “The service is funded by a per test fee from clinical commissioning groups (CCG) in 36/86 (41.9%) of services and NHS England in 1/86 (1.2%) of services. The NHS trust funded the service in 41.9% (36/86) trusts. Funding sources were unknown in 8/86 (9.3%) or other in 5/86 (5.8%) (Fig. 5)” should instead read, “The service is funded by a per test fee from clinical commissioning groups (CCG) in 12/86 (14.0%) of services and NHS England in 1/86 (1.2%) of services. The NHS trust funded the service in 60/86 (70.0%) trusts. Funding sources were unknown in 8/86 (9.3%) or other in 5/86 (5.8%) (Fig. 5)”.

In the ‘Discussion’ section, the line reading, “There has however been progress in the funding since 2011 with more than 40% now receiving fees from clinical commissioning groups” should instead read, “There has however been progress in the funding since 2011 with 84% now receiving fees from individual NHS trusts or CCGs”.
